# Supplementary material for: Arginine Expedites Erastin-Induced Ferroptosis through Fumarate
Source: Int J Mol Sci. 2023 Sep 27;24(19):14595. doi: 10.3390/ijms241914595 (PMC10572513; doi:10.3390/ijms241914595)
Supplement: Supplementary file 1 [file ijms-24-14595-s001.zip › ijms-2560896-SI.pdf]

Supplementary Figure S1

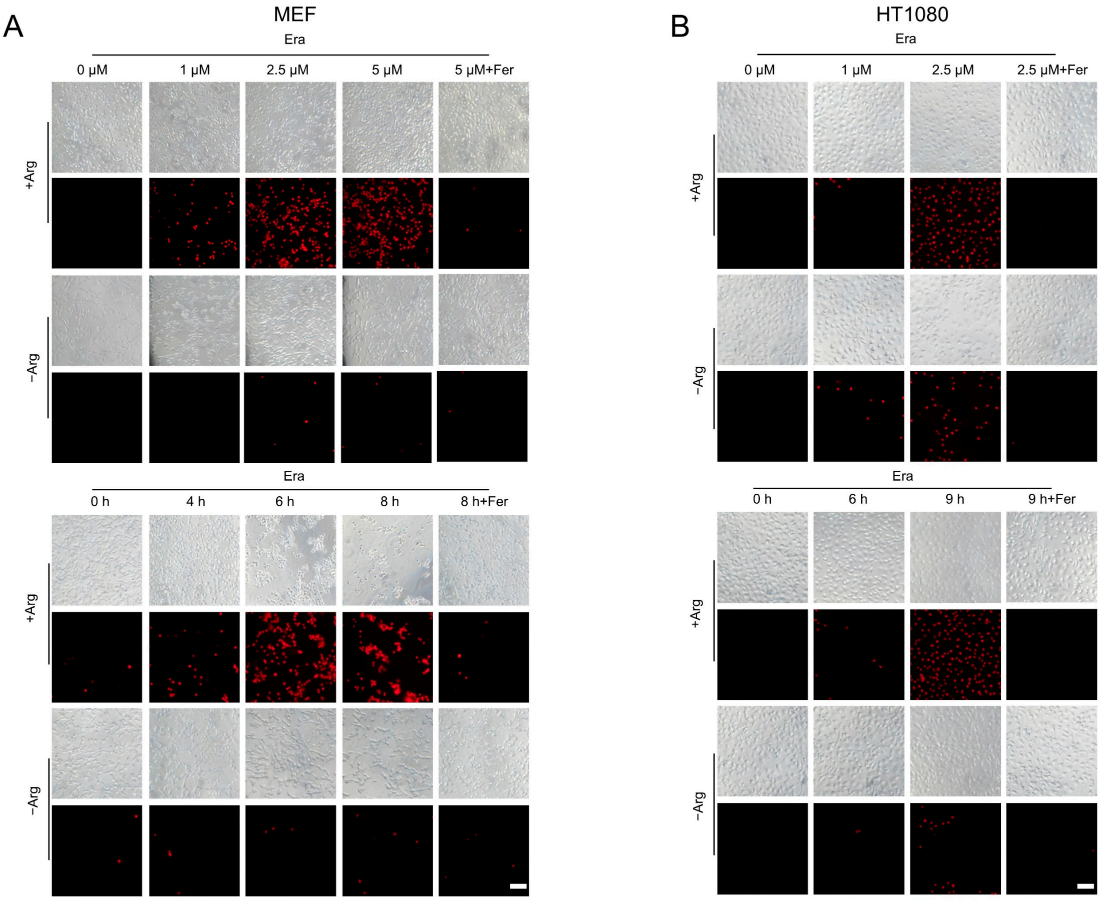

Figure S1. Arginine promotes erastin-induced ferroptosis in MEF and HT1080 cells. (A, B) MEF and HT1080 cells were treated with the indicated dose of erastin in the complete or arginine-depleted medium, with or without Fer for 6 h and 9 h, respectively, or treated with 2.5 μM erastin for the indicated time in the complete or arginine-depleted medium, with or without Fer. Cell death was visualized by PI staining and fluorescent microscopy imaging. Representative images were presented. Scale bar, 100 μm. +Arg: complete medium, −Arg: arginine-depleted medium, Era: erastin, Fer: Ferrostatin-1.
